# The road to defining African family medicine core values

**DOI:** 10.4102/phcfm.v16i1.4803

**Published:** 2024-11-18

**Authors:** Jane F. Namatovu, Innocent K. Besigye

**Affiliations:** 1 Department of Family Medicine, Makerere University, Kampala, Uganda; 2 WONCA Africa, Kampala, Uganda

## Context

The World Organization of Family Doctors (WONCA) has continued to develop and update the definition of family medicine.^1^ Family medicine training programmes have developed in the WONCA Africa Region (WAR) and many countries have progressed from pre-contemplation through to action and maintenance.^2^ There has been growth in the number of new academic family medicine programmes across the region, both within and between countries. The newly established Eastern, Central and Southern Africa College of Family Physicians (ECSA-CFP) will contribute to the scale-up of family medicine training and increase the number of family physicians within the region.^3^

Every discipline has its body of knowledge and philosophy.^4^ Therefore, as the discipline of family medicine continues to evolve, family physicians need to develop their identity as a distinct medical speciality. Understanding their identity will guide training, clinical practice and help to set the research agenda for family medicine. Studies in sub-Saharan Africa (SSA) have shown a lack of understanding of family medicine among medical students, practitioners, policymakers and the public.^5^ This occurs despite a global acceptance that strong primary care is associated with better health outcomes at a lower cost.^6,7^

An African consensus definition and principles of family medicine were described in 2008 when family medicine was present in only a few countries.^8^ As family medicine continues to develop and spread, this understanding may also evolve. However, the top 10 principles from 2008 resonate with global family medicine and are listed in Table 1.^8^ One key concept was that although we share a set of core values and principles, these are implemented differently in various countries and contexts. It is unlikely and probably undesirable that we agree on one model of care, but it should be possible to agree on the underlying values and principles.

**TABLE 1:** Top 10 principles for family medicine in Africa as defined in 2008.

Number	Principle
1	Seeing the person and their illness in relation to their context; this may be biomedical, familial, occupational, social, cultural or environmental.
2	An approach that deals with all issues related to healthcare, for all ages, sexes and regardless of the presenting problem, the organ system involved or the disease.
3	Promoting a holistic assessment, which includes biomedical, psychological, social and environmental factors – bridging the dualist distinction between mind and body, physical and psychological illness.
4	A discipline in which the specialist family physician is able to perform most of the common clinical procedures and operations appropriate to the district health system – including the district hospital – and to refer patients appropriately for procedures that are outside the scope of practice.
5	Care that is provided to a person in his or her totality and not for a specific disease or organ system.
6	A discipline in which the specialist family physician requires postgraduate training after the basic medical degree.
7	Committed to utilising resources by decision making that is evidence-based, ethical and sensitive to the personal needs of the patient, as well as equitable and fair to the community and health system.
8	A commitment to care of the person that is open-ended and not for any specific episode of illness or specific disease.
9	A speciality that is fully competent to care for all the common health problems in a specific community.
10	Care for the person in the context of their significant others, household members and family, with the provider sometimes engaging with the whole family or other groups, such as couples.

*Source:* Mash R, Moosa S, De Maeseneer J. Exploring the key principles of Family Medicine in sub-Saharan Africa: International Delphi consensus process: Open forum. S Afr Fam Pract. 2008;50(3):60–65. https://doi.org/10.1080/20786204.2008.10873720

## Project description

The WONCA World commissioned a team to describe the family medicine core values at a global level. This is being performed through regional teams to collect region-specific information on the meaning of family medicine and its core values. The Africa region team is composed of representatives from West, East and Southern Africa and has been actively working towards inputting the African voice into this project. At the 8th WAR conference in Nairobi, a workshop was held to get the views of the participants on the subject. Online information by member organisations has also been collected. Member organisations have also been contacted directly to provide relevant information that may be informative to this project.

The collected information is being collated and analysed thematically to discern the contemporary core values of family medicine within the African context. The methods, findings and conclusions will be disseminated in an upcoming WONCA book on global core values. This book will be a great resource and reference for family medicine advocates and will help in defining family medicine for governments, policymakers and managers of health systems. The book will also help researchers in defining family medicine.

## Lessons learnt so far

A clear definition and understanding of family medicine are obscured by contextual issues in SSA health systems. The contribution of family physicians to the health of people and communities needs to be clearly articulated and evidence-based, particularly in a context where most generalist doctors have no training in family medicine. First contact primary care is mostly offered by nurses and clinical officers (physician assistants) and not by family physicians. Family physicians, therefore, tend to work in multidisciplinary teams where their roles may also include capacitating the team and improving quality, as much as seeing patients. The global understanding of family physicians as providers of first-contact primary care seems not applicable within our region.

Family medicine is understood differently among member organisations within the WAR. This is evidenced by the different descriptions provided by member organisations, although the online information is sparse. Even during the workshop, there was a great debate on the meaning of family medicine among the participants. Through this project, we hope to renew the consensus on family medicine values and principles in SSA.
